# Vineyard Location Impact on the Composition and Quality of Wines from International and Native Varieties Grown in Drama, Greece

**DOI:** 10.3390/foods14071268

**Published:** 2025-04-04

**Authors:** Adriana Skendi, Aikaterini Karampatea, Elisavet Bouloumpasi, Georgia Tseine, Stefanos Stefanou, Spyridon Mamalis

**Affiliations:** 1 Department of Agricultural Biotechnology and Oenology, Democritus University of Thrace, 1st Km Dramas—Mikrohoriou, GR-66100 Drama, Greece; katerina_karampatea@yahoo.gr (A.K.); ebouloum@vo.duth.gr (E.B.); georgia_tseine@yahoo.gr (G.T.); 2 Department of Agriculture, International Hellenic University, P.O. Box 141, GR-57400 Thessaloniki, Greece; stefst2@ihu.gr; 3 Department of Management Science and Technology, Democritus University of Thrace, GR-65404 Kavala, Greece; mamalis@mst.duth.gr

**Keywords:** wine, elemental composition, phenolic compounds, antioxidant capacity, location, variety

## Abstract

The objective of this work was to investigate the effect of location on the composition and quality of wines from the viticultural zone PGI Drama. Grapes from two white (Sauvignon blanc, Assyrtiko) and three red varieties (Merlot, Cabernet Sauvignon, Agiorgitiko) were collected from nine locations within the zone during 2022. The vineyards span distances ranging from several hundred meters to 100 km, and their altitudes vary from 90 to nearly 820 m. Vinification was performed following the same protocol according to the type of wine. Wines were analyzed for quality parameters such as pH, total acidity, alcohol, and residual sugar content. In addition, elemental composition, phenolic content, antioxidant capacity, and sensory attributes of the wines were assessed. The obtained results suggested that besides the type of wine and variety, the location significantly affects the quality parameters of the wine. PCA analysis revealed that location is an important factor affecting the wine quality. The areas north and northwest proved more suitable for specific varieties, as they produce wines with more distinct organoleptic characteristics. The results provide insights into the behavior of international and native varieties in the face of global warming and assist in decisions concerning the most suitable plant material.

## 1. Introduction

According to the OIV (International Organization of Vine and Wine), Greece is the 20th largest wine-growing country in the world, producing approximately 1.3% of the total European wine [1]. There are more than 300 Greek indigenous varieties in addition to the international ones cultivated in the nine Greek wine-growing regions (mainland and islands). Each growing area is unique in terms of climate, soil composition and altitude (ranging from sea level to over 1000 m). Greece has 33 Protected Designation of Origin (PDO) and 114 Protected Geographical Indication (PGI) wines officially registered in the EU (European Union) eAmbrosia database [2]. The Drama wine-producing area in the East Macedonia and Thrace region (northeast of Greece) is recognized for its wines of high quality. Varieties such as Sauvignon blanc, Cabernet Sauvignon, Merlot, Assyrtiko, and Agiorgitiko represent 20.8%, 13.9%, 13.7%, 7.6% and 3.8%, respectively, of the approximately 510 hectares of vineyards in the region.

It is generally accepted that grapes and the wines derived from them bear characteristics linked to their place of origin [3,4]. The composition of a wine is linked to its quality and is derived from factors such as the environment, genetics and viticultural practices [5,6]. On the other hand, one aspect of the terroir concept [7] relates to the effect of the environment on wine. It comprises four factors generally accepted to affect wine quality: climate (temperature, rainfall, sunlight), soil composition (soil type, hydrology), topography (elevation, slope), and microbial terroir (in soil and facilities). Thus, terroir imparts distinctive qualities to the final wine and affects its chemical composition. Both the elemental profile and metabolites present in wine are results of terroir. Moreover, the literature reports that the composition of wine varies, even when an identical grapevine clone and/or winemaking protocol are utilized, due to differences in soil and microclimate [8].

The elemental profile of wines is of great importance not only to the wine’s physicochemical and sensory characteristics [8] but also to its origin [9,10]. Trace substances and elements have been used in the past to classify red wines according to their geographical region of origin or grape variety [10].

On the other hand, phenolic compounds play a fundamental role in the quality of wines because they contribute not only to their sensorial characteristics (color, flavor, body and structure) but also to their antioxidant capacity. The “French paradox”, which describes a reduced risk of total mortality and cardiovascular disease despite the consumption of a diet rich in cholesterol and saturated fat, is attributed to moderate red wine consumption [11]. Phenolic compounds with antioxidant capacity are present in wine and have been reported to have beneficial effects on human health. Their concentration increases as grapes ripen, but the maximum phenolic content does not coincide with maximum sugar levels [12]. Furthermore, the production of phenolics evolves differently [13]. According to Monagas et al. [14], three main factors affect the presence of phenolics in wine: grape variety (a genetic variable), region of origin (an environmental variable that includes soil type, climate, solar radiation and altitude) and the winemaking process. In particular, enological practices can play a crucial role in the final profile and amount of phenolics in the resulting wines [15,16].

The sensitivity of viticulture to climate variability is reported in the literature, with examples showing that an increase in temperature can lead to changes in grapevine phenological stages and accelerate grape maturation in some regions [17], while in others, the risk of frost might lower their occurrence [18]. These findings suggest that the use of short-term and long-term adaptation strategies is crucial in viticulture to address the impacts of climate change [19]. It is recognized that a strong relationship exists between climate and phenology, grape composition at harvest, total production, and quality. Climate change affects the ripening quality of grapes for existing varieties and wine styles, making it difficult to produce high-quality wines [20].

Regarding the vineyard environment, the migration of vineyards to higher elevation zones [21] is one of the proposed measures for climate change adaptation, along with the use of different grape varieties, either later-maturing [22] or newly developed [23], and the adaptation of vineyard management practices [24,25]. In this context, the study of different terroir zones in the Drama region of Greece is of interest, especially in relation to how the region can adapt to the challenges of climate change. Although Drama is recognized as a PGI region, no studies have been conducted to characterize the wines produced in this region or to determine what makes them unique.

Thus, this study aimed to examine the association between location and the chemical, elemental, and phenolic content, as well as sensory parameters of wines, providing insights into the impact of terroir on wine quality and organoleptic characteristics. The findings will contribute information that enables a more precise geographical classification of the Drama PGI region and offers a valuable perspective on the future cultivation of both international and native Greek grape varieties.

## 2. Materials and Methods

### 2.1. Vineyard Locations

**Figure 1:**
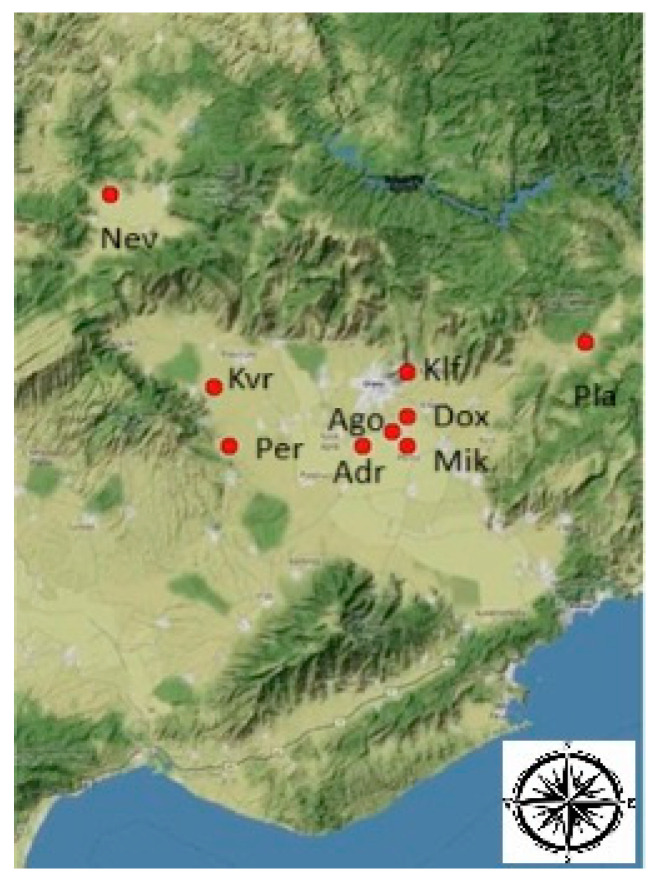
Map depicting the location of vineyards (red spots) included in this study.

The grapes were collected during the 2022 vegetative period from the vineyards located in nine sub-sones (terroir units) of the Drama regional unit of Greece (Figure 1). Vineyards were chosen based on systematic analysis, as suggested in Karapetsas et al. [26]. These vineyards are managed by five wineries, and detailed information about them is reported in Table 1. Grapes that have reached their technological maturity (according to the respective winery procedure) were manually harvested early in the morning and immediately transferred to the experimental winery of the Marketing Research and Development Laboratory for New Food and Beverage Products at the Department of Agricultural Biotechnology and Oenology of Democritus University of Thrace. The samples were processed within 24 h following the appropriate protocol, depending on the type of wine.

### 2.2. Vinification

Grapes were processed accordingly to produce respective monovarietal red and white wines. Two standardized winemaking protocols, one for the production of white wine and the other for red wine, were used to minimize variation during winemaking. White grapes from Sauvignon blanc and Assyrtiko were first hand-destemmed and then pressed using a hydraulic press (Hydro press PEW80, Grifo-Marchetti, Cremona, Italy). Sulfite (6 g/hL SO_2_) was added to the obtained must, which was allowed to clarify by static settling for 12 h at 8 °C without the addition of any other oenological products, in 30 L thermo-regulated stainless steel tanks. On the other hand, the red grapes from the varieties Merlot, Cabernet Sauvignon, and Agiorgitiko were hand-destemmed, crushed, and placed into the same type of tanks as mentioned above, where sulfiting (5 g/hL SO_2_) was performed.

Inoculation with *Saccharomyces cerevisiae* (Exelcia Terroir CH, Burgundia Oenologie, Beaune, France) at a dose of 25 g/hL was carried out once the must for both white and red wine fermentation reached 16 °C. Fermentation was performed at a controlled temperature, maintained at 15–20 °C for white and 20–30 °C for red wines. Fermentations were carried out under controlled temperature gradients, with different temperature ranges for white and red vinification. The temperature gradient was consistent throughout each phase of alcoholic fermentation. Light pressure was used to separate the wine from the pomace at the conclusion of the alcoholic fermentation process. At the end of alcoholic fermentation, which lasted at least three weeks, each wine was racked into new tanks and kept at 4 °C for 7 days with additional sulfiting (8 g/hL SO_2_ for white wines and 10 g/hL SO_2_ for red wines). Clear wine was poured into 0.75 L dark green glass wine bottles. Malolactic fermentation was deliberately avoided to reduce uncontrollable variability caused by bacterial influence on the experimental wines’ physicochemical composition and sensory qualities. Each vinification was performed in duplicate.

### 2.3. Chemical Analysis

#### 2.3.1. Chemicals and Reagents

The DPPH (2,2-diphenyl-1-picrylhydrazyl) and (+)-catechin were purchased from Sigma Aldrich (St. Louis, MO, USA), whereas TPTZ (2,4,6-tripyridyl-s-triazine) and aluminum chloride-6-hydrate were obtained from Alfa Aesar GmbH & Co (KG, Karlsruhe, Germany). ABTS (2,2′-azinobis (3-ethylbenzothiazoline-6-sulfonic acid), Trolox ((S)-(-)-6-hydroxy-2,5,7,8- tetramethylchroman-2-carboxylic acid), and gallic acid were obtained from J&K Scientific GmbH (Pforzheim, Germany). Sodium acetate trihydrate, sodium hydroxide (HPLC grade), and Folin–Ciocalteu reagent were obtained from Chem-Lab NV (Zedelgem, Belgium). Sodium carbonate, iron (III) chloride hexahydrate, and sodium nitrite were obtained from Merck KGaA (Darmstadt, Germany).

For ICP-OES analysis, only HPLC (High-Performance Liquid Chromatography) with gradient-grade methanol and water (Chem-Lab NV, Zedelgem, Belgium) was used. Single-element standard solutions at 1000 mg/L of As, Cd, Cr, Cu, Fe, Mn, Ni, Pb, Zn, K, Na, Ca, and Mg were purchased from Sigma Chem. (St. Louis, MO, USA). Appropriate protocols to avoid contamination were followed for trace metal analysis, which involved properly washing all glassware and plastic containers first with nitric acid and then with ultrapure water. All other chemicals/reagents used in this study were of analytical grade.

#### 2.3.2. Physicochemical Analysis of Wine

Reducing sugars were measured enzymatically (as the sum of glucose and fructose) according to the OIV method—MA—AS311 using the glucose/fructose kit from Steroglass S.r.l. (Perugia, Italy), total acidity (TA) according to the OIV—MA—AS313—01, pH value according to the OIV—MA—AS313—15, and alcohol content according to the OIV—MA—AS312—01A [27]. These analyses were performed in triplicate.

The color of red wines was assessed after centrifugation (10 min at 5000 rpm) by measuring absorbance at three wavelengths: 420, 520, and 620 nm. The intensity (I) and hue (T) of the wines were determined using the method of Sudraud [28], as modified by Glories [29]. Regarding the white wines, measurements were performed at wavelengths of 280 and 420 nm, as these are associated with “total phenolic index” and “browning” in white wines, respectively [30,31,32]. The applied methodology is reported in more detail by Skendi et al. [33].

### 2.4. Determination of Phenolics and Antioxidant Activity of Wines

The wine samples were centrifuged at 5000 rpm for 15 min, and the supernatants were collected and measured. Determination of total phenolic content (TPC), total flavonoid content (TFC) and the antioxidant potential of the wines using DPPH, ABTS, and FRAP (ferric reducing antioxidant power) assays was performed following the methodology reported by Skendi, Papageorgiou and Stefanou [33]. TPC was determined using Folin–Ciocalteu’s reagent assay with gallic acid as a standard, and the results were reported as milligrams of gallic acid equivalents per mL of wine (mg GAE/mL). TFC was determined using the aluminum chloride colorimetric assay with catechin as a flavonoid standard, and the results were expressed as milligrams of catechin equivalents per mL of wine (mg CATE/mL). Three assays—DPPH, ABTS, and FRAP—were used to determine the antioxidant potential of wines, using Trolox as the standard, and the results were expressed as mg Trolox equivalents per mL of wine (mg TE/mL). All the aforementioned assays were performed in at least triplicate.

### 2.5. Determination of Elemental Composition of Wines

Wines were centrifuged (15,000 rpm/min for 15 min at 5 °C), and an aliquot was placed into a water bath at 90 °C until the volume was reduced to approximately 50% of the initial volume. The wine was then reconstituted to its initial volume with 8% nitric acid. For the determination of the macroelements, a dilution factor of 10 was applied to the reconstructed samples. When necessary, the samples were diluted with 8% nitric acid before analysis. It was proved that wines tested with ICP-OES without prior treatment give results comparable to those obtained using the acid digestion procedure [33,34].

The elemental composition of the wines (macroelements: Ca, K, Mg, and Na; microelements: Cu, Fe, Mn and Zn; and trace elements: Pb, Cd, Ni, As and Cr) was determined using atomic emission spectrometry with an inductively coupled plasma source (ICP-OES), using the model 8300 DV (Perkin-Elmer, Waltham, MA, USA). To achieve better sensitivity, the determination was performed using an axial plasma view.

ICP-OES was operated at 1500 W, with a concentric quartz nebulizer with an argon gas flow of 10 L/min and an auxiliary flow of 0.6 L/min. The sample was introduced through a glass concentric nebulizer mounted on a glass cyclonic spray chamber at a speed of 0.35 L/min. The emission wavelengths (nm) used are as follows: Fe (238.204), Cu (327.393), Zn (206.200), Mn (257.610), K (766.490), Ca (317.933), Mg (285.213), Na (589.592), Cd (228.802), Pb (220.353), Ni (231.604), Cr (267.716) and As (188.979).

Calibration was performed using a three-point calibration curve, with standards for each element, taking into account the blank (ultrapure water acidified with 8% nitric acid). All calibration curves have a coefficient of determination greater than 0.9999. Satisfactory recovery and repeatability were achieved. Moreover, the wine matrix was taken into account to avoid matrix problems. All samples were analyzed in duplicate, with a blank run every 10 samples to avoid interferences/contaminations. The determination of the limit of detection (LOD) and the limit of quantification (LOQ) for the method and calibrations was performed as recommended by IUPAC [35]. The detection limits were 63.1, 79.3, 10.0, 15.0, 9.7, 1.6, 5.9, 2.6, 0.8, 2.7, 1.8, 0.9, and 4.2 μg/L for K, Ca, Mg, Na, Fe, Cu, Zn, Mn, Cd, Pb, Ni, Cr, and As, respectively. These limits ensure the determination of concentrations well below the limits set by legislation (EU, OIV) for toxic elements present in wine.

### 2.6. Sensorial Evaluation of Wines

The sensory analysis of the experimentally produced wines from grapes collected in different locations of the PGI Drama was performed after bottling by six female and four male testers (aged 27 to 50 years old). All of them were trained and experienced tasters. These panelists received appropriate training to assess the qualitative and quantitative differences between the wines. These panelists were systematically involved in wine testing. The panelists’ performance was evaluated using single-variety commercial wines of average quality and without defects from the same varieties used in the present study: Merlot, Cabernet Sauvignon, Agiorgitiko, Sauvignon blanc, and Assyrtiko. The sensorial evaluation of wine was conducted following ISO standardized methods (ISO 3591 and ISO 4121) [36,37]. The wines were presented in a random order and blind-tasted. The wine (30 mL) was tested under natural lighting in ISO wine glasses at a room temperature of approximately 20 °C and covered with plastic Petri dishes. The wine was served at an appropriate temperature for a better evaluation of its sensorial characteristics: 10–12 °C for white wines and 16–18 °C for red wines.

Wine samples were coded with three-digit numbers according to a Latin Square Design. The evaluation process included the following key sensory attributes: aroma (olfactory assessment), taste and mouthfeel (gustatory assessment), and overall harmony and quality. Each characteristic was assessed independently using a modified nine-point rating scale with anchored descriptive points for evaluating wine sensory attributes, based on the ISO 4121 method but adapted for more detailed differentiation. This approach allows for a more granular assessment of wine characteristics while still maintaining the simplicity of a five-point system. The scale was structured as follows: 1—inadequate, 1.5—less satisfactory, 2—satisfactory, 2.5—slightly below average, 3—average, 3.5—slightly above average, 4—moderately good, 4.5—very good, and 5—excellent for aroma and taste attributes; and 1—extremely poor, 1.5—very poor, 2—moderately poor, 2.5—slightly poor, 3—good, 3.5—slightly good, 4—moderately good, 4.5—very good, and 5—extremely good for overall quality of wine. The sensorial evaluation received approval from the Democritus University of Thrace Research Ethics Commission, confirming compliance with the research code of ethics.

### 2.7. Statistical Analysis

Differences were tested using analysis of variance (one-way ANOVA) followed by Duncan’s multiple range tests or the respective non-parametric Kruskal–Wallis test, depending on whether the data fulfill all the assumptions (i.e., normality, homogeneity of variance, and independence of observations). A *p*-value < 0.05 was considered significant for detecting differences between the wines. Each sample with a concentration below the LOD was assigned a value of 0. Pearson’s linear and Spearman’s correlation analyses (two-tailed, *p* < 0.05) were employed to identify any relationships between the studied parameters, depending on the type of data distribution (normal and non-normal, respectively). The software SPSS Statistics 26.0 (SPSS Inc., Chicago, IL, USA) was used to perform the abovementioned tests.

The mathematical processing of the wine data was carried out using principal component analysis (PCA) with the Pairwise estimation method. PCA analysis was performed using JMP 14 (SAS Institute Inc., Cary, NC, USA).

## 3. Results

The effect of vineyard locations within the Drama region in Greece on wine quality parameters, elemental composition, and sensorial parameters was investigated. Two white varieties (Sauvignon blanc and Assyrtiko) and three red varieties (Merlot, Cabernet Sauvignon, and Agiorgitiko) were included in the present investigation. These varieties are considered the best suited for the region and are cultivated from the five leading domains active in the area. In the present study, grapes from the Sauvignon blanc variety were collected from eight locations, whereas those from the Greek native variety Assyrtiko were collected from two. Regarding red varieties, grapes of Cabernet Sauvignon and the Greek native Agiorgitiko were obtained from three different locations, whereas those of the Merlot variety came from four locations each. The wines produced from the grapes harvested in these locations were then analyzed.

### 3.1. Physicochemical Parameters and Chromatic Characteristics of Wines

The physicochemical composition of wine, which comprises pH, total acidity, alcohol content, and residual sugars, is presented in Table 2. These parameters are considered important in winemaking, as they determine the quality of the wine. Alcohol content ranged from 9.1% vol to 12.5% vol for white wines and from 9.9% vol to 13.6% vol for red wines. Significant differences due to location are observed for each variety studied. The difference in the alcohol content of wines depends on the sugar content of the grapes, which, in turn, is strongly linked to terroir as well as different vineyard management and harvesting decisions made by wineries. A significant positive correlation (0.393, *p* < 0.05) was observed between altitude and alcohol content. This trend is contrary to the findings in the literature [38]. However, in our case, a delay of one month in ripening was observed in the higher-altitude vineyards. It caused an increased accumulation of sugar and, subsequently, elevated alcohol content.

Residual sugars depend on the fermentation process as well as the style of wine, but a dry wine contains less than 4 g/L [39]. The quantity of residual sugars varies from 0.60 to 3.5 g/L for white wines and from 0.07 to 3.6 g/L for red wines.

pH values ranged from 2.92 to 3.43 for white wines and from 3.42 to 3.99 for red wines, with the location Nevrokopi (Ne) showing the lowest values for Sauvignon blanc and Merlot wines. Nevrokopi vineyards have the highest altitude (over 800 m) among the locations. Mansour et al. [40] associated high elevation with higher levels of TA. In higher elevation sites, the nights are cooler, which helps retain acidity, balancing sugar accumulation and acid degradation. Indeed, in the present study, a significant moderate negative correlation (−0.362, *p* < 0.05) between pH and altitude was observed. Total acidity varied widely among the different varieties and locations. The presence of organic acids in wine affects its acidity [41]. Nevrokopi (Nev) showed the highest total acidity among Merlot wines.

Regarding white wines, the Mik and Kvr locations produced wines with the highest total acidity for Assyrtiko and Sauvignon blanc, respectively. Although locations such as Dox and Kvr produced Sauvignon blanc wines with high total acidity, their alcohol content was less than 10%, which can be considered an indicator of a premature harvest and is not necessarily associated with vineyard location. This was also the case for red wines produced in Mikrochori from Cabernet Sauvignon and Agiorgitiko grapes. It should be noted that harvesting was carried out by each winery according to its own scheduling and winemaking plan.

**Figure 2:**
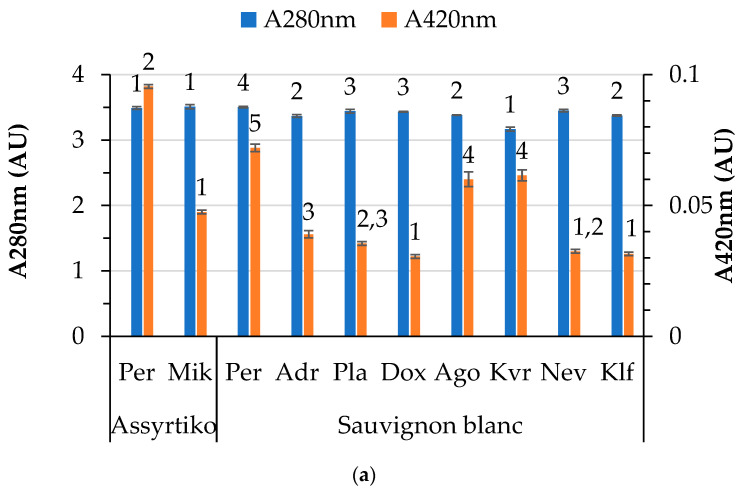

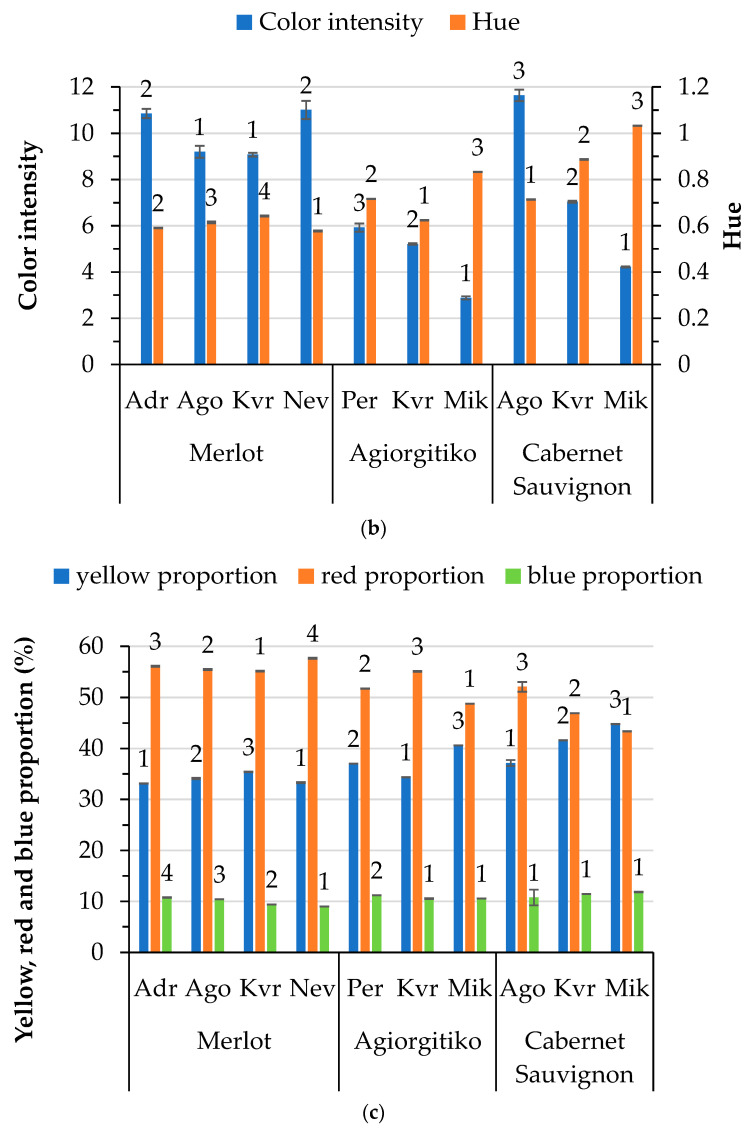
Color characteristics of wines by variety and location: (**a**) A280 nm (total phenolic index) and A420 nm of white wines; (**b**) color intensity and hue of red wines; and (**c**) percentages of yellow, red and blue hues of red wines. Abbreviations: Doxato (Dox), Adriani (Adr), Perichora (Per), Agora (Ago), Kali Vrisi (Kvr), Kallifitos (Klf), Platania (Pla), Nevrokopi (Nev), Mikrochori (Mik). Means represent results from triplicate measurements. Similar numbers above the error bars for the same parameter within the same variety are not significantly different (*p* < 0.05), according to Duncan’s multiple range test or the respective non-parametric Kruskal–Wallis test.

Variations in the color characteristics of white and red wines are presented in Figure 2. In general, significant differences are noted between locations for the same variety across all color characteristics. The A280 and A420 values for the white wines vary from 3.2 to 3.5 and from 0.03 to 0.1, respectively (Figure 2a). Different locations led to variations in the yellow color for both white varieties studied, with the Perichora (Pe) location exhibiting the highest values for both Sauvignon blanc and Assyrtiko, likely due to harvesting at a higher sugar level. A moderate positive correlation (Spearman’s rho 0.502, *p* < 0.05) was observed between yellow color intensity (A420) and pH. On the other hand, both A280 and A420 were negatively correlated with total acidity (Spearman’s rho −0.463 and −0.533, respectively, at *p* < 0.05). Organic acids (pH) may induce structural changes in phenolic compounds, leading to spectral transformations. The literature reports a pH dependence of the spectra of phenolic compounds [42].

Differences in color parameters (color intensity, hue, and proportions of yellow, red and blue) among the red wines from different locations were also noted (Figure 2b,c). It is worth noting that the blue proportion in the case of Cabernet Sauvignon was not affected by location. Differences in the color characteristics of red wines are attributed to a different level of maturity, as reported by Pérez-Magariño and González-San José [12]. These differences could be correlated with varying climatic conditions, altitude, and stages of maturation. The literature reports that different molecules (mostly anthocyanins) responsible for the color of wine are strongly affected by pH [43]. In our case, the pH was positively correlated (0.521, *p* < 0.05) only with the blue proportion in the red wines, whereas total acidity (as detailed below) was the factor most strongly affecting the color parameters. It is known that wines with low pH (high acidity) have a more red-colored hue, while lower pH values are associated with a more blueish tint [44]. Statistical analysis of the results revealed a significant positive correlation between total acidity and hue (0.463, *p* < 0.05) and yellow proportion (0.561, *p* < 0.05), and a significant negative correlation with red proportion (−0.484, *p* < 0.05). In addition, the residual sugar content is positively related to the yellow (0.535, *p* < 0.05) and negatively related to the red (−0.477, *p* < 0.05) proportions of color in red wines. Altitude is negatively related to the hue of the wine (−0.455, *p* < 0.05). Moreover, it was observed that alcohol content affects color intensity (0.813, *p* < 0.01). Increased radiation, usually observed at higher altitudes, may benefit the synthesis of anthocyanins in grape skin, which could be ascribed to the higher color intensity observed in wines from vineyards at higher altitudes [45]. Regarding the Merlot and Agiorgitiko varieties, color intensity is significantly different in wines produced at higher altitudes.

### 3.2. Variation in Phenolics and Antioxidant Capacity of Wines

**Figure 3:**
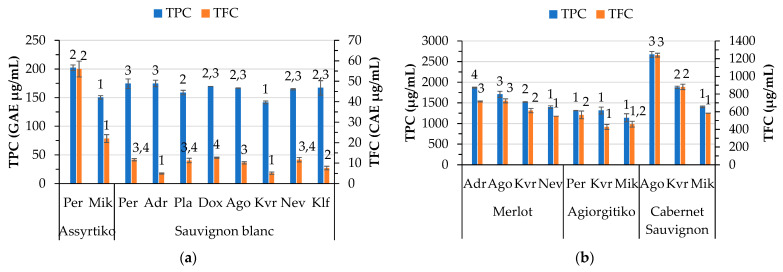

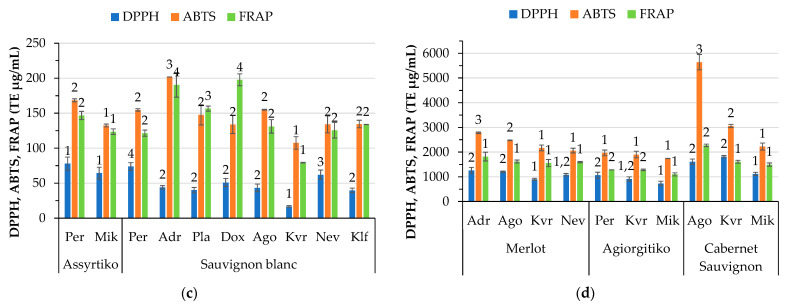
Variation by variety and location of total phenolic content (TPC GAE μg/mL), total flavonoid content (TFC CATE μg/mL) in white (**a**) and red (**b**) wines and antioxidants activity measured by DPPH, ABTS, and FRAP (TE μg/mL) for white (**c**) and red (**d**) wines. Abbreviations: Doxato (Dox), Adriani (Adr), Perichora (Per), Agora (Ago), Kali Vrisi (Kvr), Kallifitos (Klf), Platania (Pla), Nevrokopi (Nev), and Mikrochori (Mik). Means represent results of triplicate measurements. Similar numbers above the error bars for the same parameter within the same variety are not significantly different (*p* < 0.05), according to Duncan’s multiple range test or the respective non-parametric Kruskal–Wallis test.

Phenolic compounds are among the most crucial elements in wines that influence quality. The total phenolic and flavonoid content of the wines determined by spectrophotometric methods is presented in Figure 3a,b, whereas their antioxidant capacity is shown in Figure 3c,d. Locations Mik for Assyrtiko and Kvr for Sauvignon blanc wines showed the lowest phenolic content among the samples. The Assyrtiko wine made from grapes produced in Per exhibited the highest phenolic and flavonoid content. Sauvignon blanc wines from Adr and Kvr exhibited very low flavonoid content. The antioxidant activity of wines varies among the locations for each white variety, except for the DPPH antioxidant activity of Assyrtiko wines. Regarding the red wines, significant differences were observed among the locations. In the case of Cabernet Sauvignon wines, there is a more pronounced variation in the TPC and TFC among the locations compared to Merlot and Agiorgitiko. Location Agora showed the highest TPC and TFC content among all the red wines. In general, the red wines exhibited higher TPC, TFC, and antioxidant capacity than the white wines. Agiorgitiko wines did not differ in ABTS antioxidant activity across the locations. Pérez-Magariño and González-San José [12] reported that the total polyphenols in Cabernet Sauvignon wines from Spain ranged from 1951 to 2232 mg/L for the same vineyard at different maturity stages. In our case, the TPC for Cabernet Sauvignon ranged from 1403 and 2674 mg/L among locations. Kallithraka et al. [46] reported phenolic values that fall within this range for Cabernet Sauvignon but higher values for Agiorgitiko, a Greek red wine. In the literature, it is noted that there are differences in the content of phenolics among vintages [47].

In red wines, no correlation was observed between altitude and parameters such as phenolic, flavonoid content and antioxidant capacity of wine, despite the literature reporting an increase in the content of total phenolic compounds, total flavonoids and total anthocyanins with increasing altitude [48]. A very strong association (0.920, *p* < 0.01) was observed between TPC and TFC, as well as between these parameters and the antioxidant capacity of wine (DPPH, ABTS, and FRAP) (r > 0.795, *p* < 0.01), suggesting that the compounds responsible for the antioxidant activity are mainly phenolics.

Regarding the white wines, TPC was positively correlated with DPPH, ABTS, and FRAP (r > 0.453, *p* < 0.05), whereas TFC was positively correlated only with DPPH (0.775, *p* < 0.01). A high association of TFC (0.637, *p* < 0.01) and TPC (0.726, *p* < 0.01) with the color intensity of red wines suggests that flavonoids may be responsible for the enhancement of the color intensity of red wines. This finding is supported by the literature indicating that flavonols, which are yellow pigments, are mostly responsible for the color in white wines, while anthocyanins are responsible for the color in red wines [49]. Anthocyanins, mostly red pigments, outweigh the yellow flavonols in red wines [50]. Nevertheless, the flavonoids in red wines play an important role in the wine color through co-pigmentation interactions with anthocyanidins [51].

### 3.3. Elemental Composition of Wine

The results from the elemental analysis of wines are reported in Table 3 and Table 4. It is evident that the samples differ in their macro and microelement content. Statistically significant differences were observed among samples of the same variety in the same location, regardless of the element studied. Among the macro elements, potassium has the highest concentration in wines, followed by magnesium, calcium, and lastly, sodium.

In the samples analyzed, K levels ranged from 444.7 to 1951.5 mg/L (Table 3). This variation was much higher than that observed for local, homemade wines from the same region (50.3–123.2 mg/L) [33] or other Greek regions (321–1267 mg/L) [52]. In our case, the same minimal clarification process was applied to all types of wine, revealing differences among the varieties and the locations. Other than winemaking practices, potassium levels in wine are influenced by a variety of factors, such as genetic and cultural influences, environmental conditions, and vineyard management options [53].

The high variance observed for K is primarily due to grape variety, although significant differences were also observed among locations for the same variety. For example, within the same variety, Sb, grown in different locations, K values vary from 444.7 mg/L in Nev to 701.6 mg/L in Adr, while for Cabernet Sauvignon, values vary from 1054.2 mg/L in Kvr to 1951.5 mg/L in Mik. The lowest level of K was observed in the Sauvignon blanc variety, whereas the highest was found in Cabernet Sauvignon. Merlot and Agiorgitiko grape varieties have similar range levels, which are higher than those of Assyrtiko and Sauvignon blanc, which do not differ from each other, as revealed by the Kruskal–Wallis analysis test. Moreover, it was observed that red wines have a higher amount of K than white wines, a finding supported by the literature [54].

Regarding the remaining macroelements—Ca, Mg, and Na—their values vary in the ranges of 19.8–38.8 mg/L, 44.2–73.7 mg/L, and 5.8–12.9 mg/L, respectively (Table 3). In addition to K, Ca, and Mg are of great importance for yeast fermentation; however, the literature reports that higher levels of Ca may alter magnesium uptake by yeast [55]. The concentrations of macroelements K, Ca, Mg, and Na in 90 Greek wine samples originating from regions other than the one studied (across nine varieties, white and red) for two consecutive vinifications in 2017 and 2018 were reported to be 705 ± 265 mg/L, 81 ± 18 mg/L, 87 ± 17 mg/L and 23 ± 19 mg/L [56]. The levels of Ca and Mg observed in the present study showed a much lower range of variation, with values indicating the lower range of those reported in the literature for Ca [9,10] and Mg [10,57], respectively. In our study, there was no difference in the amount of Ca between the red and white wines. A similar observation was reported by Đurđić, Pantelić, Trifković, Vukojević, Natić, Tešić and Mutić [54]. On the other hand, Bora, Călugăr, Bunea, Rozsa and Bunea [9] noted that white wines have higher Ca levels than red wines. Regarding Mg, our observations are in agreement with those reported in the literature, suggesting higher concentrations in red wines than in white wines [54,58].

Sodium levels were much lower than the 80 mg/L limit set by the OIV [39]. These levels are higher than those reported for homemade wines from the same area (0.8–5.3 mg/L) [33] but lower than those reported by Kallithraka, Arvanitoyannis, Kefalas, El-Zajouli, Soufleros and Psarra [52] from a different location in Greece (2.7–187 mg/L). Although Na levels significantly differ among locations for the same grape variety, there are no differences among the Assyrtiko, Sauvignon blanc, Merlot, and Cabernet Sauvignon wines. Nevertheless, all of them have significantly higher Na levels than the Agiorgitiko wines. No significant difference in the levels of red and white wines was observed in our case, while Đurđić, Pantelić, Trifković, Vukojević, Natić, Tešić and Mutić [54]. However, it was noted that white wines from specific Serbian regions contain higher levels than red wines. The sodium content of the white wine may slightly increase when sodium bentonite is used during the fining process [59]. High levels of Na are primarily related to proximity to the sea and are most commonly observed in wines produced on islands [52,60].

Regarding microelements, iron showed the highest concentration, followed by zinc, magnesium, and copper (Table 3). Although present in much lower levels than macroelements, the presence of microelements is considered important for wine quality. The OIV has set limits for residues of Cu (1 mg/L) and Zn (5 mg/L) [39], while recommending that Fe levels be below 10 mg/L. The levels of microelements vary in the range of 0.931–2.974 mg/L, <LOQ 0.376 mg/L, 0.578–0.923 mg/L, and 0.226–1.263 mg/L for Fe, Cu, Zn, and Mn, respectively. Pasvanka, Kostakis, Tarapoulouzi, Nisianakis, Thomaidis and Proestos [56] reported Fe, Cu, Zn, and Mn values of 0.86 ± 0.56 mg/L, 87 ± 50 mg/L, 0.52 ± 0.23 mg/L, and 1.3 ± 0.43 mg/L, respectively, in 90 Greek wines. In our study, we observed visibly higher levels of Fe, lower levels of Cu and Mn, and similar values of Zn compared to other wine samples from different regions of Greece. On the other hand, wines from Crete had higher levels of Fe (4.7–12 mg/L), Zn (0.3–31 mg/L), and Mn (0–10 mg/L) than the wines in the present study, but showed similar Cu values (0.2–0.6 mg/L) [61]. Moreover, the Fe and Cu levels observed in our study are similar to those reported for Romanian wines, whereas Zn and Mn levels are lower [9]. All microelement concentrations (Fe, Cu, Zn, and Mn) are lower than those reported in Serbian wines [54]. It has been reported that elemental composition in wine can be influenced by winemaking treatments [62]. In our case, all wines were produced following a similar procedure (for red and white wines, respectively), minimizing differences due to the procedures applied. All wines showed values significantly lower than the limits set by the OIV for Cu and Zn and the recommendations for Fe. There are significant differences in the concentrations of these microelements in wines produced from grapes of the same variety but grown in different locations. Previous research has indicated a clear relationship between the elemental profile of wines and the mineral composition of vineyard soils [63]. Additionally, grape variety appears to be a significant factor affecting the quantity of microelements in wine. In this study, the Assyrtiko variety showed the lowest Fe content, whereas the highest levels were found in the Merlot and Agiorgitiko wines. The lowest levels of Mn were observed in Assyrtiko wines, while no difference was observed among the other varieties. The lowest level of Cu was observed in Agiorgitiko, whereas the highest was observed in Assyrtiko. Both Assyrtiko and Merlot showed the lowest levels of Zn, whereas the Sauvignon blanc wines contained the highest Zn levels. In the present study, red wines showed higher levels of Fe and Mn than white wines, whereas no differences were observed in the Cu and Zn levels. Other researchers have also noted the effect of vine variety on the mineral composition of grapes and wines, as a result of variations in the absorption of metals from the soil [64].

In the present study, no sample surpassed the OIV limits for Cd (0.01 mg/L), Pb (0.15 mg/L), and As (0.2 mg/L) [39] or the limit set by the EU for Pb (0.1 mg/kg) [65]. As was not detected in our samples, whereas the Pb and Cd values were below the abovementioned limits (Table 4). Cr levels vary from below the LOD to 11.39 μg/L, whereas the values of Ni range from 61.38 to 152.45 μg/L. In this study, Pb and Cr were higher in red wines than in white wines, whereas the opposite was observed for Ni levels. No differences were observed in the amount of Cd between the red and white wines. The findings regarding Pb are similar to those found by Đurđić, Pantelić, Trifković, Vukojević, Natić, Tešić and Mutić [54] but not to those regarding Ni and Cr. The literature reports different potential factors that influence the levels of toxic metals in grapes, such as soil composition, pesticide application and environmental pollution [66]. In wine, these contaminants may originate from the grapes or be introduced during the winemaking process through prolonged contact with equipment and other operations [67].

### 3.4. Sensorial Analysis of Wine

**Figure 4:**
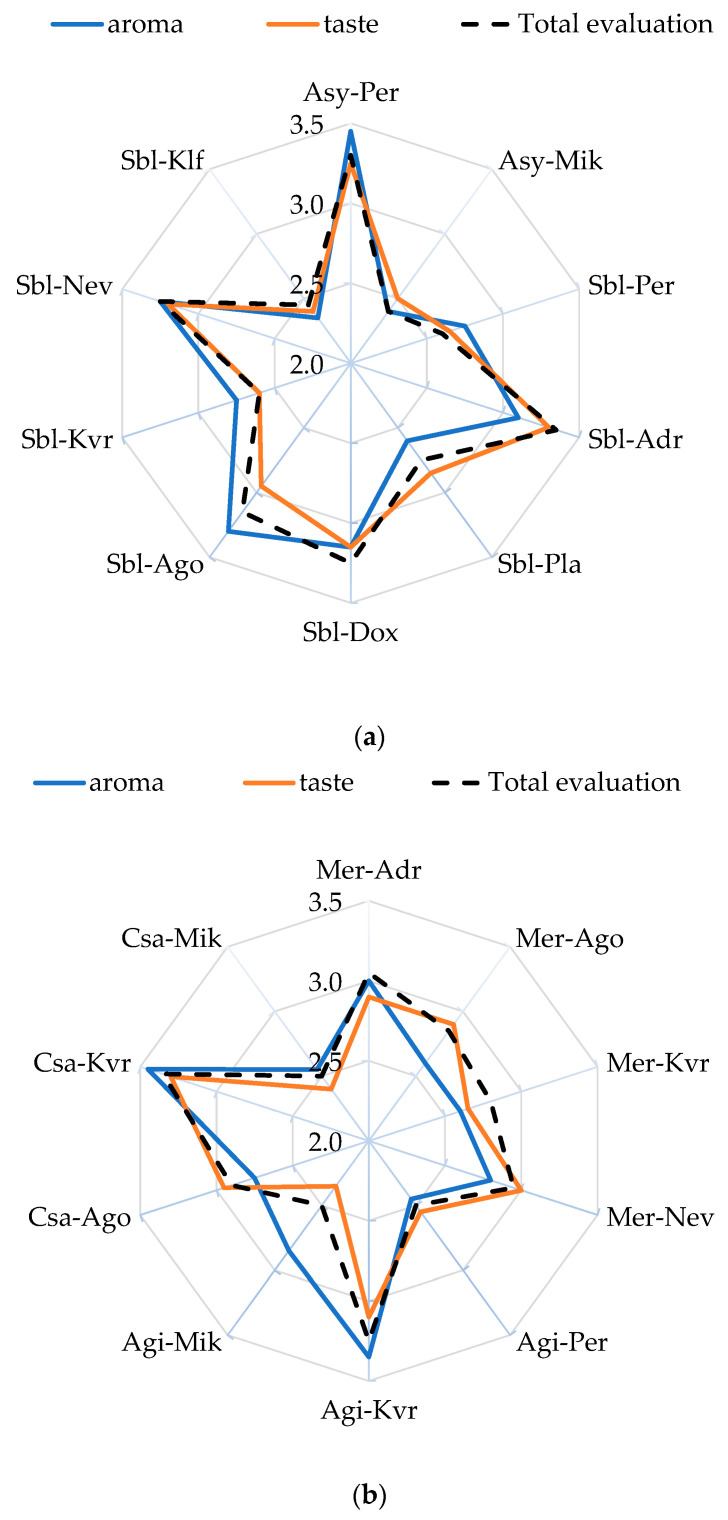
Sensory evaluation (mean values) of (**a**) white and (**b**) red wines in different locations. Abbreviations: grape variety: Assyrtiko (Asy), Sauvignon blanc (Sbl), Merlot (Mer), Agiorgitiko (Agi), Cabernet Sauvignon (Csa); locations: Doxato (Dox), Adriani (Adr), Perichora (Per), Agora (Ago), Kali Vrisi (Kvr), Kallifitos (Klf), Platania (Pla), Nevrokopi (Nev), and Mikrochori (Mik).

Figure 4 shows the average scores reflecting the level of perceived aroma and taste attributes, as well as overall acceptance. The organoleptic evaluation of the produced wines revealed that the location of the grapes contributed to the formation of wines that differ in aroma, taste, and overall score. For the Assyrtiko white wines, both aroma and taste were rated as “slightly below average” for the Mikrochori location, which also received a “slightly poor” rating for overall quality. In contrast, wines for Perichora were rated “slightly above average” for aroma and taste and “slightly good” for overall quality. In the case of Sauvignon blanc, the aroma of the wines from Ago and Nev and the taste of wines from Adr were evaluated as “slightly above average”. On the other hand, Sauvignon blanc wines from Kallifitos and Kali Vrisi locations received the lowest scores for both aroma and taste. Wines from Pla, Dox, and Ago did not differ in taste and were rated as “average”. In general, the overall acceptability of wines from Klf, Kvr, and Per was “very poor”, whereas wines from Adr, Dox, and Nev received a “slightly good” score.

Regarding red wines, location plays a significant role in the aroma and taste of the wines. In the case of the Merlot variety, although no significant difference was observed in the total evaluation of the wine among the locations, differences were noted in aroma and taste (considered “good”). The aroma of wines from Ago and Kvr was evaluated as “slightly below average”, as was the taste of wines from Kvr. The Mikrochori location produced Cabernet Sauvignon wine with the lowest ratings for aroma, taste, and overall acceptability, a trend that was also observed for Agiorgitiko from the Perichora location. In the case of Agiorgitiko and Cabernet Sauvignon, the wines from Kvr stood out in terms of overall acceptability.

It seems that in the case of red wines, altitude correlated well with taste (0.858, *p* < 0.01) and overall acceptability (0.681, *p* < 0.05) of the wines. The results suggest that the red varieties perform better at higher altitudes. Malinovski et al. [68] suggest that Cabernet Sauvignon performs better at higher altitudes, as it needs very cold conditions during the winter period. On the other hand, in the case of white wines, no correlation was found between altitude and the characteristics of the wine. Although the polyphenol composition of wine affects its sensory properties [69], in our study, it does not correlate significantly with TPC and TFC.

A positive correlation exists between alcohol content and the sensorial attributes of taste and overall acceptance (regardless of the type of wine). An increase in alcohol content is linked with an increase in taste (0.554, *p* < 0.05) and overall acceptance (0.474, *p* < 0.01) in wines. No significant association was found for white wines, whereas in red wines, a correlation was observed only with taste (0.754, *p* < 0.05). This trend is contrary to the findings in the literature, which report that fruity aroma decreases with increasing levels of alcohol [70]. In general, sensorial attributes did not show significant correlations with total acidity, residual sugars, or pH of the wine, even when the type of wine was considered. Although these parameters are deemed crucial to the quality of wine, wine is a very complex matrix, and the levels as well as the interactions among its components affect the overall perceived sensorial quality of wine.

### 3.5. PCA Analysis of the Data

**Figure 5:**
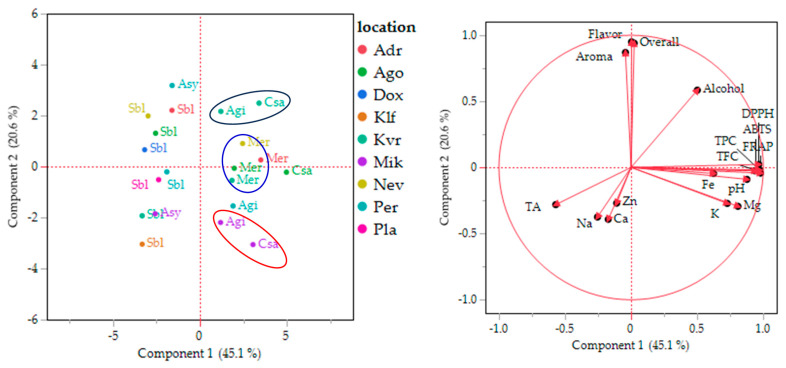
Plots of principal component analysis scores (**left**) and loadings (**right**) for the composition of the wines.

PCA analysis revealed that principal component 1 (PC1) and principal component 2 (PC2) explain 65.7% of the total variance—45.1% and 20.6%, respectively (Figure 5). Figure 5 (right) illustrates the variables that make significant contributions to the formation of PC1 and PC2. PC2 comprises all parameters related to the “organoleptic” characteristics of wine, including flavor, overall acceptability, aroma, alcohol content, and the concentrations of Ca, Na, and Zn. All the PC2 factors are directly related to the impact of fermentation on organoleptic characteristics. On the other hand, the remaining parameters are grouped under the PC1 factor. These factors are mostly related to the wine quality parameters, including phenolic content, antioxidant capacity, pH, and the concentrations of Fe, K, and Mg, which indirectly influence the “color stability” of wine. The literature reports that anthocyanins and tannins can form complexes with metals such as Fe, Cu, Al, and Mg, resulting in changes in wine color [71].

PC1 was negatively affected only by total acidity (TA), while PC2 was influenced by the concentrations of Ca, Na, and Zn. Indeed, wines with low acidity are more prone to oxidation, which affects the “color stability” component [43]. In addition, Na gives a salty taste to wine, while Ca and Zn affect yeast growth and the production of secondary metabolites, thereby affecting the aroma and taste, the “organoleptic” components of wine [72,73].

In Figure 5 (left), all observations in the dataset are labeled with their respective variety and denoted in different colors according to their location. The white wines are located in the negative part of the PC1 component, while the red wines are in the positive part, indicating that color is the main discriminant among the wines. The second factor is location: wines from the two varieties, Agiorgitiko and Cabernet Sauvignon, cultivated in the same locations (Mik and Kvr), are closer to each other than to other samples belonging to the same variety from different locations. This pattern reinforces the fact that, for these two varieties, location has a greater influence on wine quality than the grape variety. On the other hand, Merlot samples are grouped together regardless of location, indicating higher stability of this variety across different locations.

The collected data led to a better understanding of the influence of terroir on the five grapevine varieties studied within the relatively small geographical area of PGI Drama. The physicochemical parameters, phenolic content, and antioxidant capacity have been linked with elemental composition, providing the opportunity to obtain information on geographical origin. In the present study, the effect of processing on wine quality parameters has been minimized, as the same vinification procedure was applied to each type of wine (white and red). This helped reveal the effect of location.

## 4. Conclusions

Our study shows that location can significantly influence wine characteristics. It seems that in addition to the vineyard altitude influencing the quality and organoleptic characteristics of the wines produced, the location also affects the elemental composition of the wine. Significant differences were observed between red and white wines, with red wines produced at the highest altitudes receiving the highest scores for aroma, taste, and overall acceptability. This study demonstrates the potential for distinguishing between wines based on their geographical origin utilizing trace element profiles, phenolic content, and antioxidant capacity data.

The present data highlight the vast variability within a relatively small viticulture region, providing a valuable basis for making decisions on agronomic and plant material selection as part of climate change mitigation strategies. Our study provides valuable information for winemakers seeking to adapt to climate change and highlights the importance of strategic plant material choices to mitigate the effects of future global warming. Moreover, the recent interest in considering altitude when developing strategies to delay grape ripening and ensure it occurs at lower temperatures stems from its role as a key viticultural factor in reducing the negative effects of global warming.

Nevertheless, the results of this study are based on only one cultivation season and should not be generalized. Additionally, the projected increase in temperature and CO_2_ levels in the atmosphere in the coming years is expected to require an increased water supply. Given the predicted changes in environmental conditions, higher-altitude regions may become more suitable for producing high-quality wines. Yet, the influence of other specific factors, such as the choice of plant material, training systems, and water availability, must be considered.

## Figures and Tables

**Table 1:** Descriptive information regarding the location and samples involved in this study.

Sample Code	Grape Variety	Vineyard Location	Vineyard Coordinates	Altitude (m)	Winery
Sb-Do	Sauvignon blanc (Sbl)	Doxato (Dox)	41°07′00″ N/24°14′39″ E	100	W1
Sb-Ad		Adriani (Adr)	41°07′23.1″ N/4°15′15.3″ E	160	W2
Sb-Pe		Perichora (Per)	41°05′39.6″ N/23°56′57.4″ E	180	W3
Sb-Ag		Agora (Ago)	41°07′36.4″ N/24°16′18.5″ E	180	W4
Sb-Kv		Kali Vrisi (Kvr)	41°09′43.0″ N/23°53′48.9″ E	220	W5
Sb-Kl		Kallifitos (Klf)	41°11′30.9″ N/24°11′27.0″ E	280	W5
Sb-Pl		Platania (Pla)	41°11′59.8″ N/24°25′37.9″ E	350	W2
Sb-Ne		Nevrokopi (Nev)	41°22′18.5″ N/23°41′43.0″ E	820	W2
As-Mi	Assyrtiko (Asy)	Mikrochori (Mik)	41°06′18.2″ N/24°10′10.7″ E	80	W5
As-Pe		Perichora (Per)	41°05′41.9″ N/23°56′55.4″ E	180	W3
Me-Kv	Merlot (Mer)	Kali Vrisi (Kvr)	41°09′04.8″ N/23°55′25.5″ E	150	W2
Me-Ad		Adriani (Adr)	41°07′10.2″ N/24°14′44.3″ E	150	W2
Me-Ag		Agora (Ago)	41°07′33.6″ N/24°16′21.7″ E	190	W4
Me-Ne		Nevrokopi (Nev)	41°22′07.5″ N/23°41′32.7″ E	860	W2
Cs-Mi	Cabernet Sauvignon (Csa)	Mikrochori (Mik)	41°06′38.0″ N/24°11′11.4″ E	100	W5
Cs-Ag		Agora (Ago)	41°07′38.0″ N/24°16′35.3″ E	190	W4
Cs-Kv		Kali Vrisi (Kvr)	41°10′00.9″ N/23°53′20.6″ E	200	W4
Ag-Mi	Agiorgitiko (Agi)	Mikrochori (Mik)	41°06′27.6″ N/24°10′51.6″ E	100	W5
Ag-Pe		Perichora (Per)	41°05′41.9″ N/23°56′49.8″ E	190	W3
Ag-Kv		Kali Vrisi (Kvr)	41°09′25.7″ N/23°54′22.0″ E	210	W2

**Table 2:** Physicochemical parameters of the wines from different locations of Drama region *.

Color	Variety	Location	pH		Total Acidity (g Tartaric Acid/L)		Alcohol (% Vol.)		Residual Sugars (g/L)	
white	Asy	Per	3.37 ± 0.03	^1^	6.45 ± 0.04	^1^	12.30 ± 0.14	^2^	3.00 ± 0.35	^1^
		Mik	3.31 ± 0.02	^1^	7.36 ± 0.02	^2^	9.10 ± 0.03	^1^	3.40 ± 0.02	^1^
	Sbl	Per	3.23 ± 0.02	^4^	7.66 ± 0.03	^2^	12.50 ± 0.04	^8^	2.40 ± 0.02	^5^
		Adr	3.43 ± 0.01	^6^	6.70 ± 0.01	^1^	12.00 ± 0.07	^6^	2.70 ± 0.01	^6^
		Pla	3.25 ± 0.01	^4^	8.41 ± 0.03	^4^	11.10 ± 0.01	^5^	0.60 ± 0.03	^1^
		Dox	3.16 ± 0.01	^3^	10.50 ± 0.02	^7^	9.60 ± 0.02	^1^	3.50 ± 0.01	^8^
		Ago	3.30 ± 0.02	^5^	8.33 ± 0.01	^3^	10.20 ± 0.03	^4^	3.30 ± 0.02	^7^
		Kvr	3.14 ± 0.02	^3^	10.85 ± 0.03	^8^	9.70 ± 0.03	^2^	1.80 ± 0.03	^3^
		Nev	2.92 ± 0.02	^1^	10.00 ± 0.02	^6^	12.40 ± 0.03	^7^	0.90 ± 0.01	^2^
		Klf	3.03 ± 0.01	^2^	9.00 ± 0.02	^5^	10.00 ± 0.02	^3^	2.30 ± 0.01	^4^
red	Mer	Adr	3.82 ± 0.01	^3^	5.97 ± 0.02	^1^	12.10 ± 0.04	^2^	0.07 ± 0.00	^1^
		Ago	3.58 ± 0.01	^2^	6.65 ± 0.02	^2^	11.60 ± 0.04	^1^	0.60 ± 0.01	^2^
		Kvr	3.61 ± 0.01	^2^	6.66 ± 0.02	^2^	12.10 ± 0.04	^2^	0.10 ± 0.01	^1^
		Nev	3.42 ± 0.02	^1^	8.02 ± 0.026	^3^	13.60 ± 0.04	^3^	2.90 ± 0.01	^3^
	Agi	Per	3.45 ± 0.01	^1,2^	7.31 ± 0.017	^2^	11.20 ± 0.03	^3^	1.70 ± 0.02	^2^
		Kvr	3.42 ± 0.03	^1^	6.93 ± 0.011	^1^	10.90 ± 0.04	^2^	1.10 ± 0.01	^1^
		Mik	3.53 ± 0.03	^2^	7.82 ± 0.028	^3^	9.90 ± 0.03	^1^	3.60 ± 0.02	^3^
	Csa	Ago	3.73 ± 0.01	^1^	7.60 ± 0.012	^2^	12.70 ± 0.05	^2^	3.10 ± 0.01	^3^
		Kvr	3.68 ± 0.02	^1^	7.01 ± 0.031	^1^	13.50 ± 0.04	^3^	1.80 ± 0.02	^1^
		Mik	3.99 ± 0.03	^2^	8.45 ± 0.032	^3^	10.30 ± 0.03	^1^	2.70 ± 0.02	^2^

* Results are reported as means ± standard deviation (SD) of three replicates. Total acidity is expressed in mg/L tartaric acid equivalent. Means with similar superscript numbers in the same column within the same variety are not significantly different (*p* < 0.05), according to Duncan’s multiple range test or the respective non-parametric Kruskal–Wallis test.

**Table 3:** Macro- and microelements in wines ^1^.

Color	Variety	Location	K		Ca		Mg		Na	
**white**	**Asy**	Per	542.4 ± 0.3	^2^	22.5 ± 0.2	^1^	50.5 ± 0.3	^2^	9.12 ± 0.14	^1^
		Mik	511.5 ± 3.5	^1^	25.9 ± 0.0	^2^	47.9 ± 0.1	^1^	9.86 ± 0.01	^2^
	**Sbl**	Per	541.2 ± 3.8	^5^	22.0 ± 0.2	^1^	55.2 ± 0.5	^7^	8.26 ± 0.05	^2^
		Adr	701.6 ± 1.5	^7^	22.5 ± 0.0	^1^	51.1 ± 0.1	^5^	10.23 ± 0.03	^4^
		Pla	599.6 ± 14.0	^6^	26.4 ± 0.1	^2^	50.5 ± 0.2	^4,5^	10.26 ± 0.05	^4^
		Dox	504.5 ± 1.2	^3^	32.4 ± 0.6	^4^	48.7 ± 0.7	^2^	11.30 ± 0.26	^5^
		Ago	592.2 ± 1.3	^6^	33.0 ± 0.1	^5^	44.2 ± 0.1	^1^	7.02 ± 0.02	^1^
		Kvr	471.4 ± 6.5	^2^	35.0 ± 0.1	^6^	52.9 ± 0.5	^6^	12.17 ± 0.15	^6^
		Nev	444.7 ± 2.9	^1^	28.3 ± 0.3	^3^	50.0 ± 0.5	^3,4^	8.74 ± 0.08	^3^
		Klf	521.2 ± 11.3	^4^	38.8 ± 0.3	^7^	49.3 ± 0.2	^2,3^	12.91 ± 0.20	^7^
**red**	**Mer**	Adr	925.2 ± 66.0	^3^	32.6 ± 0.0	^3^	63.3 ± 0.1	^3^	9.36 ± 0.02	^2^
		Ago	794.1 ± 6.0	^2^	33.1 ± 0.0	^4^	53.3 ± 0.2	^1^	9.73 ± 0.02	^3^
		Kvr	786.4 ± 6.5	^2^	30.6 ± 0.1	^2^	57.7 ± 0.2	^2^	9.32 ± 0.05	^2^
		Nev	685.1 ± 4.2	^1^	26.2 ± 0.0	^1^	66.0 ± 0.5	^4^	8.16 ± 0.05	^1^
	**Agi**	Per	771.3 ± 5.1	^2^	23.5 ± 0.1	^1^	68.5 ± 0.6	^2^	7.16 ± 0.07	^2^
		Kvr	711.7 ± 8.4	^1^	26.8 ± 0.1	^2^	59.1 ± 0.3	^1^	5.84 ± 0.03	^1^
		Mik	854.7 ± 4.7	^3^	29.9 ± 0.1	^3^	73.7 ± 0.2	^3^	8.82 ± 0.01	^3^
	**Csa**	Ago	1204.8 ± 6.9	^2^	32.6 ± 0.1	^3^	65.3 ± 0.3	^2^	9.07 ± 0.09	^1^
		Kvr	1054.2 ± 17.3	^1^	19.8 ± 0.0	^1^	63.6 ± 0.5	^1^	11.26 ± 0.20	^2^
		Mik	1951.5 ± 27.1	^3^	24.1 ± 0.1	^2^	73.0 ± 0.5	^3^	10.91 ± 0.08	^2^
**color**	**variety**	**location**	**Fe**		**Cu**		**Zn**		**Mn**	
**white**	**Asy**	Per	1.043 ± 0.016	^2^	0.037 ± 0.006	^1^	0.629 ± 0.008	^1^	0.226 ± 0.002	^1^
		Mik	0.931 ± 0.010	^1^	0.376 ± 0.001	^2^	0.715 ± 0.003	^2^	0.230 ± 0.001	^1^
	**Sbl**	Per	1.034 ± 0.003	^2^	<LOQ	^1^	0.923 ± 0.010	^4^	0.360 ± 0.002	^2^
		Adr	1.153 ± 0.000	^5^	0.250 ± 0.002	^3^	0.862 ± 0.021	^3^	0.341 ± 0.002	^1,2^
		Pla	1.150 ± 0.009	^5^	0.046 ± 0.000	^1^	0.848 ± 0.017	^3^	0.312 ± 0.004	^1^
		Dox	1.258 ± 0.003	^7^	0.014 ± 0.005	^1^	0.785 ± 0.011	^2^	0.657 ± 0.011	^4^
		Ago	1.114 ± 0.003	^4^	<LOQ	^1^	0.750 ± 0.014	^1^	0.312 ± 0.004	^1^
		Kvr	1.175 ± 0.006	^6^	0.143 ± 0.061	^2^	0.749 ± 0.005	^1^	0.912 ± 0.043	^6^
		Nev	1.022 ± 0.003	^1^	<LOQ	^1^	0.856 ± 0.007	^3^	0.708 ± 0.003	^5^
		Klf	1.050 ± 0.001	^3^	0.011 ± 0.013	^1^	0.851 ± 0.004	^3^	0.594 ± 0.003	^3^
**red**	**Mer**	Adr	2.616 ± 0.012	^3^	<LOQ	^1^	0.844 ± 0.010	^3^	0.470 ± 0.006	^1^
		Ago	1.435 ± 0.002	^1^	0.096 ± 0.010	^2^	0.578 ± 0.011	^1^	0.583 ± 0.013	^2^
		Kvr	1.643 ± 0.020	^2^	0.150 ± 0.004	^3^	0.698 ± 0.008	^2^	0.724 ± 0.003	^3^
		Nev	2.974 ± 0.021	^4^	0.189 ± 0.008	^4^	0.688 ± 0.030	^2^	0.817 ± 0.017	^4^
	**Agi**	Per	2.193 ± 0.012	^3^	<LOQ	^1^	0.888 ± 0.007	^3^	1.263 ± 0.005	^3^
		Kvr	1.690 ± 0.001	^1^	0.007 ± 0.001	^2^	0.671 ± 0.008	^1^	0.542 ± 0.002	^2^
		Mik	1.844 ± 0.010	^2^	<LOQ	^1^	0.834 ± 0.010	^2^	0.456 ± 0.003	^1^
	**Csa**	Ago	1.391 ± 0.018	^2^	<LOQ	^1^	0.751 ± 0.009	^1^	0.426 ± 0.003	^2^
		Kvr	1.233 ± 0.002	^1^	0.109 ± 0.001	^2^	0.843 ± 0.009	^2^	0.705 ± 0.005	^3^
		Mik	1.469 ± 0.009	^3^	0.004 ± 0.002	^1^	0.864 ± 0.010	^2^	0.360 ± 0.007	^1^

^1^ Results (in mg/L) are reported as means ± standard deviation (SD) of three replicates; <LOQ—below the limit of quantification. Abbreviations: grape variety: Assyrtiko (Asy), Sauvignon blanc (Sbl), Merlot (Mer), Agiorgitiko (Agi), and Cabernet Sauvignon (Csa); locations: Doxato (Dox), Adriani (Adr), Perichora (Per), Agora (Ago), Kali Vrisi (Kvr), Kallifitos (Klf), Platania (Pla), Nevrokopi (Nev), and Mikrochori (Mik). Means with similar superscript numbers in the same column within the same variety are not significantly different (*p* < 0.05), according to Duncan’s multiple range test or the respective non-parametric Kruskal–Wallis test.

**Table 4:** Trace elements in wines ^1^.

Color	Variety	Location	Cd		Pb		Ni		Cr	
**white**	**Asy**	Per	<LOD		<LOD		152.45 ± 0.81	^2^	<LOD	
		Mik	<LOD		<LOD		76.95 ± 3.93	^1^	<LOD	
	**Sbl**	Per	8.66 ± 0.05	^5^	47.35 ± 3.63	^5^	94.42 ± 0.08	^4^	<LOD	
		Adr	3.59 ± 0.30	^3^	17.63 ± 0.86	^3^	91.45 ± 0.78	^3,4^	<LOD	
		Pla	3.99 ± 0.25	^4^	11.08 ± 0.36	^2^	78.99 ± 4.18	^1^	<LOD	
		Dox	<LOD		<LOD		84.57 ± 1.94	^1,2^	<LOD	
		Ago	<LOD		<LOD		83.33 ± 1.21	^1,2^	<LOD	
		Kvr	<LOD		<LOD		87.41 ± 2.99	^2,3^	<LOD	
		Nev	2.19 ± 0.11	^1^	27.14 ± 3.16	^4^	95.55 ± 1.89	^4^	<LOD	
		Klf	2.71 ± 0.05	^2^	4.90 ± 0.32	^1^	90.61 ± 3.10	^3,4^	<LOD	
**red**	**Mer**	Adr	7.94 ± 0.06	^2^	64.90 ± 0.90	^3^	74.39 ± 2.05	^2^	7.11 ± 0.48	^3^
		Ago	<LOD		36.68 ± 0.97	^1^	86.63 ± 0.22	^3^	1.91 ± 0.09	^1^
		Kvr	1.37 ± 0.09	^1^	56.99 ± 2.37	^2^	61.38 ± 2.18	^1^	3.33 ± 0.31	^2^
		Nev	1.34 ± 0.37	^1^	<LOD		119.82 ± 1.94	^4^	<LOD	
	**Agi**	Per	8.13 ± 0.83	^2^	39.98 ± 8.52	^1^	68.26 ± 0.65	^1^	2.31 ± 0.12	^1^
		Kvr	1.03 ± 0.00	^1^	<LOD		81.01 ± 0.67	^2^	<LOD	
		Mik	1.77 ± 0.40	^1^	40.85 ± 0.25	^1^	110.11 ± 0.22	^3^	11.39 ± 0.21	^2^
	**Csa**	Ago	1.39 ± 0.32	^1^	48.19 ± 1.08	^1^	75.73 ± 1.24	^2^	1.44 ± 0.30	^1^
		Kvr	7.10 ± 0.57	^2^	58.90 ± 0.83	^2^	70.74 ± 0.86	^1^	1.08 ± 0.16	^1^
		Mik	0.96 ± 0.12	^1^	<LOD		72.66 ± 0.67	^1^	<LOD	

^1^ Results (in μg/L) are reported as means ± standard deviation (SD) of three replicates; <LOD—below the limit of detection. Abbreviations: grape variety: Assyrtiko (Asy), Sauvignon blanc (Sbl), Merlot (Mer), Agiorgitiko (Agi), and Cabernet Sauvignon (Csa); locations: Doxato (Dox), Adriani (Adr), Perichora (Per), Agora (Ago), Kali Vrisi (Kvr), Kallifitos (Klf), Platania (Pla), Nevrokopi (Nev), and Mikrochori (Mik). Means with similar superscript numbers in the same column within the same variety are not significantly different (*p* < 0.05), according to Duncan’s multiple range test or the respective non-parametric Kruskal–Wallis test.

## Data Availability

The original contributions presented in this study are included in the article, further inquiries can be directed to the corresponding author.
